# The Effect of Postoperative Physical Therapy Following Hip Fracture: A Literature Review

**DOI:** 10.7759/cureus.37676

**Published:** 2023-04-17

**Authors:** Smaragda Koudouna, Dimitrios S Evangelopoulos, Michail Sarantis, Efstathios Chronopoulos, Ismene A Dontas, Spiros Pneumaticos

**Affiliations:** 1 Department of Physiotherapy, General Hospital of Attika 'KAT', Athens, GRC; 2 3rd Department of Orthopedic Surgery, National and Kapodistrian University of Athens, 'Attikon' University Hospital, Athens, GRC; 3 4th Department of Orthopedic Surgery, General Hospital of Attika 'KAT', Athens, GRC; 4 Department of Orthopedic Surgery, Laboratory for Research of the Musculoskeletal System (LRMS) of the School of Medicine, University of Athens, Athens, GRC; 5 Department of Veterinary Medicine, Laboratory for Research of the Musculoskeletal System (LRMS) of the School of Medicine, University of Athens, Athens, GRC; 6 Department of Veterinary Medicine, General Hospital of Attika 'KAT', Athens, GRC; 7 Department of Veterinary Medicine, National and Kapodistrian University of Athens, 'Attikon' University Hospital, Athens, GRC

**Keywords:** eq-5, sf-36, cci, hhs, tug, spbb, physical therapy, rehabilitation, hip fracture

## Abstract

Hip fractures in the elderly have become a major public health concern as the population ages. Post-operative rehabilitation is associated with improved outcomes and a greater likelihood of returning to pre-operative functional capacity. Several studies have been conducted to investigate various post-operative recovery pathways. However, little is known about which post-operative rehabilitation pathways for hip fractures are most effective in improving patient outcomes. No clear evidence-based guidelines for a standard mobilization protocol for patients are currently available. This review aims to investigate post-operative recovery pathways to help patients suffering from hip fracture return to pre-fracture condition and to quantify pre-operative and post-operative scores for objective rehabilitation evaluation. Measuring pre-operative activity and comparing it to post-operative follow-up values can help predict post-operative rehabilitation functional outcomes.

## Introduction and background

Hip fragility fractures are a common occurrence in the elderly [1]. Their prevalence is increasing globally as life expectancy rises, with an incidence rate of 350/100,000 people per year in developed countries [1]. Despite medical advances and increasingly sophisticated therapeutic interventions, hip fractures continue to have a significant impact on elderly patients (reduced mobility and functionality) [2]. They are associated with significant social [3] and economic consequences (increased hospitalization costs and nursing home rates, high mortality and morbidity) [4,5]. Many patients who have had a hip fracture are unable to return to their pre-fracture level of autonomy [5]. Age, presence of comorbidities, lack of autonomy prior to the fracture, and prolonged bed rest (frequent use of an air pressure mattress, an increase in the number of days spent disoriented, inability to recover, long-term urinary catheter, and short-term care provision) are all risk factors [6]. Prolonged immobility can decrease functionality and raise the risk of hospital-related problems like falls and pressure ulcers. In contrast, early movement tends to decrease functional decline and bed rest comorbidities. According to current literature, following the core concepts of an early mobilization protocol is related to better patient outcomes, such as a shorter hospital stay and maintenance or improvement of functional status from admission to discharge [7]. Receiving post-operative rehabilitation is related to better results and a higher likelihood of returning to the previous functional status. Many studies have investigated various post-operative rehabilitation strategies. Early post-operative improvement has been demonstrated to occur with quick and thorough care following hip surgery, and early mobilization improves early recovery. However, little is known about which post-operative rehabilitation pathways are most useful in improving patient outcomes. There are no clear evidence-based guidelines for a standard mobilizing procedure for patients with hip fractures. Therefore, the current study aimed to evaluate the effects of post-operative physical therapy following hip fracture in the acute phase during the hospital stay and during the outpatient post-operative period.

Patient evaluation tools

The primary goal of post-operative rehabilitation is to help patients return to their pre-fracture condition. Therefore, quantifying pre-operative and post-operative scores is required to evaluate treatment objectively. Measuring pre-operative activity and comparing it with post-operative follow-up values can help predict the outcomes of post-operative rehabilitation and the effectiveness of therapeutic protocols to improve mobility and function in patients after hip fracture surgery. Several useful scoring systems are available in the international literature to score the condition of patients during their hospital stay. In addition, the scores can be monitored continuously after discharge. Health-related quality of life (HRQOL) may be measured using several generic questionnaires, such as the SF-36 and EQ 5-VAS, commonly used in studies to measure health quality [8,9]. After a hip fracture, the patient's quality of life in terms of physical activity and social and emotional well-being suffers dramatically. Most patients improved within six months of the fracture but did not return to their pre-fracture clinical condition [8]. Comorbidities, post-operative discomfort, duration of hospital stay, and complications were all significantly linked with health status and quality of life. Patients with total or semi-arthroplasty had better health than those with internal osteosynthesis [8]. Recurrent falls in the elderly are connected with lower limb weakness [10]. The Short Physical Performance Battery test (SPPB) is one of the most popular tests used to measure lower limb strength. Many studies have shown that it is a relevant and accurate prognostic tool for evaluating senior mobility, function, and physical performance [10,11]. It is also beneficial for assessing the risk of falls, impairments, nursing home admissions, and death. Its validity was demonstrated by its association with various health, mobility, and functional ability measures. It is safe, simple, and standard, making it suitable for use in clinical practice and research. Furthermore, because the grading criteria are suited for an aged patient group, they may be applied safely and reliably in hospitalized senior patients [10]. The SPPB is a combination of metrics that combines the outcomes of maintaining balance, gait speed, and lower limb strength endurance (chair stand test) [11]. Each sector is evaluated with a maximum of four points, resulting in a total score that runs from 0 (lowest performance) to 12 (best performance). The Timed Up and Go (TUG) test provides a score that assesses functional mobility by measuring the time (in seconds) it takes a person to stand up from a chair, walk three meters on a line painted on the floor and return to the chair [12]. The test has been utilized in several studies worldwide and helps predict rehabilitation outcomes by quantifying functional mobility and predicting the risk of falls [13]. Patients who are not permitted to bear full weight postoperatively or are unable to walk prior to the fracture (walking aids are permitted but without help) may be excluded. The TUG test is performed the day before hospital discharge and during follow-up at the outpatient clinics. The Harris Hip Score (HHS) is the most used instrument for assessing hip disorders. It was created to assess the outcomes of hip surgery and the associated impairments in adult populations. It is scored on a scale of 0-100 and assesses four components (pain, functionality, deformity, and movement). The pain section (0-44 points) assesses the intensity of pain, its impact on daily activities, and the requirement for pain relievers. The functioning portion (0-47 points) is divided into everyday activities (using a ladder, using public transportation, sitting in a chair, wearing shoes and socks) and gait (limping, use of a walking aid, and gait distance). The deformity section (deformation = 0 points, absence of deformation = 4 points) investigates hip flexion, adduction, internal rotation, and extremity length discrepancy. The range of motion section (ROM) (0-5 points) measures the hip's range of motion by scoring six motions based on the maximal ROM. The overall score is determined by adding the points from the four areas. A total score of less than 70 is regarded as bad, 70 to 79 as average, 80 to 89 as good, and above 90 as an excellent result [14]. The Charlson Comorbidity Index predicts mortality, mobility, and post-operative complications following a hip fracture [15,16]. In a research by Cecchi F et al. [17], moderate-to-high comorbidity and communication problems were found to be common findings that also indicate recovery failure in patients with hip fractures who receive intense inpatient rehabilitation. At the time of admission, 97% of patients admitted to the ICU and 64% admitted to the non-ICU had moderate-to-severe comorbidity [17]. Poor outcome was associated with older age, higher comorbidity, urinary catheter use, imbalance, impaired cognitive and functional status at admission, and pre-fracture disability. However, only higher comorbidity and impaired communication on admission predicted failure to recover functionality at discharge [17]. The main goal of this review is to investigate previous published medical literature about post-operative recovery pathways to help patients suffering from hip fracture return to pre-fracture condition and to quantify pre-operative and post-operative scores for objective rehabilitation evaluation. 

## Review

Rehabilitation at the hospital

Early Mobilization

Early mobilization is essential for the best post-operative care of patients with hip fractures. Getting the patient out of bed and sitting on it, getting out of bed and sitting in a chair, getting up from the chair, and walking with walking assistance are all examples of early mobility [18-22]. The recommendations of Australia's and New Zealand's National Institutes of Health and Care (NICE) and Australian and New Zealand Hip Fracture Registry (ANZHFR) encourage mobilization the day following surgery unless medically or surgically contraindicated or if the patient was unable to walk prior to the fracture [21,23]. Early aided movement (within 48 hours of surgery) hastens functional rehabilitation, is related to faster hospital release and return home, and results in fewer admissions to high-quality rehabilitation and care units [24]. Partial or full weight-bearing early mobility of patients contributes to early recovery. Partial weight-bearing is critical for activating osteoblasts and other bone repair cells. Furthermore, surgical immobility may cause further difficulties. Non-weight bearing on the operated limb when walking, according to biomechanical rules, shifts the center of gravity away and increases the pressures imposed on the abductor's muscles, resulting in compressive forces many times the body weight [25]. As a result, mild weight-bearing, or even toe-touch weight-bearing, is preferable to non-weight bearing. Many studies anticipate partial weight-bearing on the lower extremities following a time of non-weight bearing in an effort to generate the ideal mechanical environment at various phases of fracture healing. Partial weight-bearing entails gradually increasing the weight load on the injured limb over time, which differs across patients depending on the extent of the damage and the surgeon's discretion [26]. However, research indicates that the traditional approach of post-operative partial weight-bearing, beginning at 200 N and progressively progressing to full weight, has significant limitations in clinical practice [27]. The surgeon's decision to take a cautious approach to weight-bearing in relation to the patient's tolerance influences partial weight-bearing (post-operative pain and fatigue). Furthermore, it is uncertain how much weight produces the optimum therapeutic benefit and what way of educating patients to comply with the therapist's recommendations is the most successful. Patients' ability to perform partial weight bearing is dependent on their ability to understand and reproduce the therapist's orders, who should provide clear and comprehensive instructions on how much weight to carry to the member (verbal teaching method, demonstration, hand-under-foot method, use of biofeedback devices) [28-31].

Physiotherapy in the acute post-operative care phase

In the first post-operative weeks, patients lose more than half of their muscular strength in the operated limb compared to the healthy limb, especially in hip fractures [32-35]. Many studies [24, 36-38] suggest rapid physical treatment in the acute care phase because it enhances recovery compared to delayed physical therapy (beginning on the third or fourth day after surgery). Rapid healing from a hip fracture requires rapid weight bearing on the afflicted limb and muscular strengthening. The duration and intensity of physiotherapy in older patients with hip fracture is associated with clinical improvement and distinguishes between quick and gradual rehabilitation [28]. In the acute phase of the hospital, patients who followed a high-intensity physical therapy program (three daily sessions) reached a functionality level sufficient for hospital discharge 10 days earlier than the control physiotherapy group (one daily session), with patients reporting good adherence to the program [37]. However, high-intensity physiotherapy regimens cannot be administered to every patient in the acute phase following hip fracture surgery. Several exercises are highly suggested during the acute care phase following surgery. After surgery, patients can begin ankle pump exercises (dorsal and plantar flexion of the ankle joint). When the fracture is in the acute phase, exercise to strengthen the quadriceps and enhance knee strength at the fractured extremity is possible. Kronborg L et al. [39] investigated the effect of a daily progressive knee strengthening program on the broken limb. The patient sits with their legs dangling out of bed and uses an ankle cuff to conduct knee extensions (three sets of 10 repetitions with maximum load). There were no documented harmful effects or discomfort related to hip fracture, and muscular insufficiency after the fracture decreased from 50% to 32% on average [39]. Furthermore, other quadriceps workouts (isometric exercises and knee extension from a supine posture with a cushion under the knee) and gluteal muscle exercises result in greater muscular strength and limb control (isometric exercises of the gluteus in a supine position and pelvic lift).

Multidisciplinary management

The outcome of rehabilitation programs is dependent on an integrated interdisciplinary approach. Interdisciplinary teams examine and maximize senior individuals' medical, psychosocial, and physical capacities, including early release plans. Because most hip fractures occur in the elderly, a multidisciplinary rehabilitation plan incorporating geriatricians and other experts is critical. To get the best outcomes, this management should begin as soon as the patient is admitted to the hospital and continue throughout the patient's recuperation after release. According to the NICE and ANZHFR guidelines, orthogeriatric examination and pre-operative medical optimization of surgical capability are recommended. It is critical to identify specific goals for interdisciplinary rehabilitation care as soon as possible. Regaining mobility and freedom, as well as facilitating return home and long-term success, should all be considered. In addition, collaboration with post-operative care services, such as mental health, fall prevention, and osteoporosis management, should be explored [20,22]. Recent prospective research found that multidisciplinary treatment for older patients with hip fractures improved functional rehabilitation, decreased subsequent fractures, and lowered hip fracture care costs, lowering the economic and social burden [40]. In patients with hip fractures, Nordström P et al. [41] discovered that multidisciplinary therapy improves physical function and mobility much more than traditional care. However, it has no effect on the patient's chances of being discharged and returning home or surviving. A recent systematic review and meta-analysis of randomized controlled trials [42] assesses the impact of occupational therapy on patients' activities of daily living and total physical function (physical function, perception and sense of health, new falls) following hip fracture surgery. Because occupational therapy appears to enhance general functionality and perceptions of health and emotions, it can be included in complete rehabilitation programs following hip fracture surgery.

Physiotherapy and rehabilitation after discharge

Many patients with hip fractures restore their gait and balance abilities within the first 6-9 months after surgery [43]. Most patients are discharged from the hospital during this time and live at home (followed up as outpatients) or in rehabilitation institutions [43-45]. Following surgery, physiotherapy in the subacute phase focuses on increasing safe mobility and improving muscular function. Physical activity has been shown to minimize functional limits and impairment in healthy older adults [26-28]. According to a recent review, strength training with gradually increasing resistance can successfully enhance physical function in the elderly (enhancing muscular strength and performance of fundamental functional tasks) (Activities of Daily Living [ADL]) [29]. Lee SY et al. [46] conducted a meta-analysis to assess the efficacy of progressive resistance training following hip fracture surgery. Progressive resistance training improved the treated patients' total mobility more than the control group, especially mobility linked to ADL, balance, and lower limb strength. Knee flexion/extension, hip flexion/extension, leg press, and hip abduction exercises are used in rehabilitation regimens [46]. The workout components (intensity, number of repetitions per set, and number of sets) vary slightly from study to study. Small groups, unpredictable outcomes, and a variety of follow-up times were identified as limitations in this study. As a result, large-scale randomized controlled studies are required to quantify exercise program outcomes and assist consensus building properly. During the bone healing period (6 months to 2 years) at the fracture site, more emphasis should be placed on increasing the intensity of physiotherapy and other training, in addition to progressive resistance exercise (balance and endurance exercises, functional activities).

Supervised home-based treatment

Another critical concern in hip fracture rehabilitation programs is the efficacy of home-based remedial exercise treatment. Many studies [47-49] have suggested that home exercise is preferable to mainstream treatment. Several of them show that people with hip fractures can improve their functioning. The most effective programs are actually extensions of regular rehabilitation, with strict monitoring and frequent visits from therapists. Adding substantial physical therapy would be problematic, given the expense rise following acute care. Furthermore, the findings are contentious and require additional exploration through additional research and clinical application.

After typical recuperation, Latham NK et al. [47] employed programs advising patients to practice exercise regimens at home several times weekly for six months. Sitting and standing up from a chair and climbing and descending stairs were among the workouts. Physiotherapists came to the patients' homes and taught them the necessary rehabilitation exercises, and made monthly phone calls. Participants were also given DVD copies of these presentations [47].

In terms of functional mobility, the intervention group outperformed the control group significantly (mean SPPB score, mean Activity Measure for Post-Acute Care (AM-PAC) mobility and mean daily AM-PAC activity score). Multiple analyses revealed that the differences between the groups remained significant for SPPB and AM-PAC daily activity but not for mobility. All functional tests showed significant changes between groups at nine months. A functional home-oriented exercise program resulted in modest improvement in physical function at six months in patients who had completed normal therapy following a hip fracture [47].

Two meta-analyses reached opposing findings on these programs. According to Kuijlaars IA et al. [48], there is little evidence to demonstrate the advantage of home exercise treatment following a hip fracture for the majority of functional outcomes and activities. Wu D et al. [49], on the other hand, observed that home rehabilitation had a substantial favorable effect on physical function following hip fractures. The later meta-analysis includes papers from more recent years than the former. However, given that the techniques and measurements of the results differed, it is unclear which is more accurate.

Long-term exercise programs to prevent falls

One of the primary aims of hip fracture therapy is to restore functional levels to pre-fracture levels while also preventing falls and future fractures. Despite this, half of patients do not achieve their prior degree of autonomy and mobility [6]. Routine post-operative care is frequently insufficient to maximize recovery efficiency [50] since balance and gait recovery can take up to nine months, and gait speed improvement can take up to 11 months [51].

There is considerable interest in researching the effects of a remedial exercise program for individuals with hip fractures returning to the community after a standard healing time. Extensive exercise programs appear to increase functional capacity [52] and reduce or reverse impairment in individuals with hip fractures [50,53], whereas other exercise programs performed at home appear to improve physical fitness [52,54]. However, the outcomes of such a long-term program are unknown.

Long-term studies found that reducing rehabilitation services reduced the functional status of patients with hip fractures [55,56]. Relative immobility at home upon discharge increases muscular weakening and loss of balance, increasing the risk of subsequent falls and fractures. Binder EF et al. [57] discovered that physical function, quality of life, and disability improved considerably in the intervention group compared to the control group in a six-month outpatient rehabilitation experiment comprising gradually increasing resistance training.

In a systematic assessment of 12 studies evaluating exercise treatments in patients with a hip fracture, Resnik B et al. [58] found insufficient support for exercise's usefulness in completing ADL. Furthermore, Handoll HH et al. [59] concluded that there was insufficient data to create a clear approach for improving mobility following hip fracture surgery. Tinetti ME et al. [60] conducted a study that included patients following their return home who followed a one-year rehabilitation program with strengthening and functional exercises and found no meaningful benefits for the prevention of falls.

Finally, a recent study [61] investigated the effect of the Otago Exercise Programme (OEP) on limb function recovery in elderly patients with hip arthroplasty for a femoral neck fracture. The OEP is a common program for the prevention of falls in the elderly. The study indicates that the OEP can effectively promote limp stability and hip function recovery in elderly patients with hip arthroplasty for femoral neck fractures, improve daily mobility and quality of life, and is suitable for clinical application.

Rehabilitation of patients with vulnerability (frailty/fragility)

Physical exercise programs have improved functional outcomes such as gait speed and SPPB score in older persons with vulnerability indicators [62]. However, the consequences on quality of life [63] or ADL balance and functioning [62] are unknown. Exercise therapies have been shown to be useful in decreasing or avoiding weakness in the elderly living in the community [64]. People who show indicators of weakness tend to benefit from multi-factor exercise regimens, although the appropriate program content is unknown [65-67].

Exercise with individual instruction and supervision was more successful in improving physical function in vulnerable persons than group exercise [63]. Treatment programs must endure at least three months; programs lasting more than five months outperform those lasting less time [67]. Similar results for patients with hip fractures show that customized, multifactorial, and gradual therapy improves functional ability [50,53].

Singh NA et al. [68] studied the impact of a training program that combined high-intensity resistance exercises and balance exercises with the concurrent targeted multidisciplinary treatment of vulnerability (with interventions for osteoporosis, diet, vitamin D/calcium levels, depression, cognitive deficits, home safety, polypharmacy, hip protection, self-efficacy, and social support), mortality, nursing home admissions, functional independence, and the use of walking tubs.

Compared to the control group, the intervention group's mortality risk was lowered by 81%, admissions to nursing homes were reduced by 84%, functional independence dropped less, and the usage of walking aids was much lower at 12 months. Functional independence was connected with improvements in strength, balance, nutrition, depressive symptoms, vision, cognitive impairments, self-efficacy, and degree of habitual activity. Reduced admissions to nursing homes were linked to improved eyesight, capacity to conduct daily activities (ADL), and gait [68].

Rehabilitation of patients with sarcopenia

Sarcopenia is a frequent geriatric illness characterized by a slow decrease of skeletal muscle mass and muscular function [69], which was initially reported by Rosenberg IH [70]. Sarcopenia is related to adverse health outcomes (falls, disability, nursing home admission, and mortality). Several studies show an increasing epidemiological, biological, and clinical link between vulnerability, sarcopenia, osteoporosis, and falls, but there are no clear diagnostic criteria or treatment strategies in clinical practice [71].

In order to establish a more successful rehabilitation regimen, elderly adults with a history of fractures or falls should be examined for sarcopenia using an integrated therapeutic approach to the prevention and treatment of fragility fractures. However, few research studies have been conducted to date on sarcopenia in the elderly with a hip fracture in rehabilitation programs.

Recent research by Lim SK et al. [72] found a significant link between sarcopenia and falls in the elderly with a hip fracture. Furthermore, in a study of elderly patients admitted to rehabilitation centers, Malafarina V et al. [73] discovered that sarcopenic patients had nearly double the mortality risk compared to non-sarcopenic patients following hip fractures.

Landi F et al. [74] conducted research on the relationship between sarcopenia and functional results in senior hip fracture patients admitted to a rehabilitation center. Compared to non-sarcopenic individuals, patients with sarcopenia (33.9% of the sample) had a substantially greater risk of impaired functioning and a poorer Barthel score when discharged from the rehabilitation unit and after three months of follow-up. Because the study found that sarcopenia has a negative impact on functional rehabilitation, it is necessary to systematically evaluate sarcopenia in elderly people with hip fractures who are participating in rehabilitation programs and develop personalized treatment protocols to improve functional outcomes.

According to a recent research study [75], physical activity (resistance exercises) and nutritional supplements (mostly protein) are the primary methods for avoiding and correcting sarcopenia in older people following hip surgery. Prolonged high-intensity resistance training increases muscle growth in the elderly, improves muscular strength and functioning, and aids in hip fracture recovery.

Another recent study by Oh MK et al. [76] compared the effectiveness of a combined training program of an anti-gravity treadmill and conventional rehabilitation after a hip fracture in patients with sarcopenia. They found that both groups improved after the intervention. However, the benefits (in walking ability, Berg Balance Scale, Mini-Mental State Examination, EQ-5D questionnaire, Barthel index, and grip strength) were greater in the intervention group.

Rehabilitation of patients with cognitive deficits

Patients with cognitive impairment had more difficulty recovering from hip fractures [77]. Around 19% of older persons with hip fractures have dementia, and up to 40% have some type of cognitive impairment that does not match the conventional dementia criteria (delirium, moderate cognitive impairment) [78]. Rösler A et al. [79] established the Cognitive Geriatric Unit (CGU), a dedicated geriatric ward for patients with hip fractures and dementia. Compared to traditional geriatric therapy, the CGU provides extra benefits (hidden exit doors, increased light in corridors and patient rooms, night lights, and ward treatment room to reduce patient transport). Treatment in a dedicated CGU appears to result in improved mobility in dementia patients regardless of duration of stay. Body weight-supported treadmill training (BWSTT) was employed by Bellelli G et al. [80] for the post-operative rehabilitation of an older woman with severe cognitive impairment who underwent surgery following a hip fracture. They also assisted the patient in maintaining an upright posture and a regular walking pattern. Walking on the treadmill was done daily, with the patient's endurance increasing regularly. As functional improvement occurred, the body weight suspension was lowered, and the length of exercise increased, eventually reaching 40 minutes without weight support till release. Furthermore, before discharge, the patient was able to rise up from a chair on her own, maintain balance without assistance, and walk a few meters with assistance. After six months, functional performance was maintained [80]. According to research, BWSTT may allow for recurrent gait practice and reduce the time it takes to begin training. It offers symmetrical weight support from the lower extremities and can alleviate discomfort in the afflicted leg, resulting in increased comfort and confidence in walking abilities. BWSTT may alleviate anxiety in patients with severe dementia who do not cooperate with the standard treatment due to fear of falling. Furthermore, it might help individuals with cognitive deficiencies overcome the challenge of learning new complicated motor methods (usage of walking aids) [81-84]. BWSTT has been utilized in trials of patients following total hip arthroplasty, hemiparesis after spinal cord injury, Parkinson's disease, and heart failure, improving gait and balance more than conventional training [81-84]. If these findings can be replicated in more extensive studies, it could prove to be an interesting technique for rehabilitating patients with hip fractures and severe dementia. Although the current findings originate from a small number of research with quality issues, it should be mentioned that rehabilitation programs for persons with cognitive impairment can be implemented [85,86]. A lack of consensus on the elements to be included in rehabilitation programs, the absence of a unified approach to actively engaging members of this group in rehabilitation, inconsistency in assessing cognitive impairment, and the differentiation of cognitive impairment from delirium and depression are all limitations for more advanced studies [85].

## Conclusions

In patients with a hip fracture, post-operative rehabilitation can enhance clinical results and quality of life. However, there is insufficient evidence to document this possible advantage due to the variety of study techniques and differences in outcomes between published studies. Both measurement approaches (self-reported and performance-based) produced similar and acceptable results in terms of validity, sensitivity to change, and responsiveness. One of the primary purposes of acute geriatric units is to maintain functional abilities in the elderly patient. As a result, the advantages of frequently monitoring the functional state of elderly patients might be considerable. It is critical for orthopedists and physiotherapists to use rehabilitation procedures for these patients and grasp the advantages and disadvantages of the numerous potential solutions.

## References

[1] Kanis JA, Odén A, McCloskey EV, Johansson H, Wahl DA, Cooper C (2012). A systematic review of hip fracture incidence and probability of fracture worldwide. Osteoporos Int.

[2] Kammerlander C, Gosch M, Kammerlander-Knauer U, Luger TJ, Blauth M, Roth T (2011). Long-term functional outcome in geriatric hip fracture patients. Arch Orthop Trauma Surg.

[3] Smrke D, Biscević M (2008). Hip fracture: personal, family and social problem of the third age. Acta Med Croatica.

[4] Braithwaite RS, Col NF, Wong JB (2003). Estimating hip fracture morbidity, mortality and costs. J Am Geriatr Soc.

[5] Schaller F, Sidelnikov E, Theiler R (2012). Mild to moderate cognitive impairment is a major risk factor for mortality and nursing home admission in the first year after hip fracture. Bone.

[6] Morri M, Chiari P, Forni C (2018). What factors are associated with the recovery of autonomy after a hip fracture? A prospective, multicentric cohort study. Arch Phys Med Rehabil.

[7] Pashikanti L, Von Ah D (2012). Impact of early mobilization protocol on the medical-surgical inpatient population: an integrated review of literature. Clin Nurse Spec.

[8] Peeters CM, Visser E, Van de Ree CL, Gosens T, Den Oudsten BL, De Vries J (2016). Quality of life after hip fracture in the elderly: a systematic literature review. Injury.

[9] Scholten AC, Haagsma JA, Steyerberg EW, van Beeck EF, Polinder S (2017). Assessment of pre-injury health-related quality of life: a systematic review. Popul Health Metr.

[10] Fujita K, Nakashima H, Kako M (2020). Short physical performance battery discriminates clinical outcomes in hospitalized patients aged 75 years and over. Arch Gerontol Geriatr.

[11] Guralnik JM, Simonsick EM, Ferrucci L (1994). A short physical performance battery assessing lower extremity function: association with self-reported disability and prediction of mortality and nursing home admission. J Gerontol.

[12] Podsiadlo D, Richardson S (1991). The timed "Up & Go": a test of basic functional mobility for frail elderly persons. J Am Geriatr Soc.

[13] Kristensen MT, Foss NB, Kehlet H (2009). Factors with independent influence on the 'timed up and go' test in patients with hip fracture. Physiother Res Int.

[14] Söderman P, Malchau H, Herberts P (2001). Outcome of total hip replacement: a comparison of different measurement methods. Clin Orthop Relat Res.

[15] González-Zabaleta J, Pita-Fernandez S, Seoane-Pillado T, López-Calviño B, Gonzalez-Zabaleta JL (2016). Comorbidity as a predictor of mortality and mobility after hip fracture. Geriatr Gerontol Int.

[16] Hasan O, Barkat R, Rabbani A, Rabbani U, Mahmood F, Noordin S (2020). Charlson comorbidity index predicts postoperative complications in surgically treated hip fracture patients in a tertiary care hospital: retrospective cohort of 1045 patients. Int J Surg.

[17] Cecchi F, Pancani S, Antonioli D (2018). Predictors of recovering ambulation after hip fracture inpatient rehabilitation. BMC Geriatr.

[18] Parker M, Johansen A (2006). Hip fracture. BMJ.

[19] Mak JC, Cameron ID, March LM (2010). Evidence-based guidelines for the management of hip fractures in older persons: an update. Med J Aust.

[20] (2020). Scottish Intercollegiate Guidelines Network (SIGN) Management of hip fracture in older people. A national clinical guideline Edinburgh: SIGN. https://www.sign.ac.uk/our-guidelines/management-of-hip-fracture-in-older-people/.

[21] Ftouh S, Morga A, Swift C (2011). Management of hip fracture in adults: summary of NICE guidance. BMJ.

[22] Swierstra BA, Vervest AM, Walenkamp GH (2011). Dutch guideline on total hip prosthesis. Acta Orthop.

[23] (2014). Australian and New Zealand Hip Fracture Registry (ANZHFR) Steering Group. Sydney: ANZHFR Steering Group.

[24] Oldmeadow LB, Edwards ER, Kimmel LA, Kipen E, Robertson VJ, Bailey MJ (2006). No rest for the wounded: early ambulation after hip surgery accelerates recovery. ANZ J Surg.

[25] van Lieshout R, Pisters MF, Vanwanseele B, de Bie RA, Wouters EJ, Stukstette MJ (2016). Biofeedback in partial weight bearing: usability of two different devices from a patient's and physical therapist's perspective. PLoS One.

[26] Schoene D, Wu SM, Mikolaizak AS, Menant JC, Smith ST, Delbaere K, Lord SR (2013). Discriminative ability and predictive validity of the timed up and go test in identifying older people who fall: systematic review and meta-analysis. J Am Geriatr Soc.

[27] Vasarhelyi A, Baumert T, Fritsch C, Hopfenmüller W, Gradl G, Mittlmeier T (2006). Partial weight bearing after surgery for fractures of the lower extremity--is it achievable?. Gait Posture.

[28] Bakker A, Blokhuis TJ, Meeks MD, Hermens HJ, Holtslag HR (2014). Dynamic weight loading in older people with hip fracture. J Rehabil Med.

[29] Kamel HK, Iqbal MA, Mogallapu R, Maas D, Hoffmann RG (2003). Time to ambulation after hip fracture surgery: relation to hospitalization outcomes. J Gerontol A Biol Sci Med Sci.

[30] Yu S, McDonald T, Jesudason C, Stiller K, Sullivan T (2014). Orthopedic inpatients’ ability to accurately reproduce partial weight bearing orders. Orthopedics.

[31] Maguire C, Sieben JM, Scheidhauer H, Romkes J, Suica Z, de Bie RA (2016). The effect of crutches, an orthosis TheraTogs, and no walking aids on the recovery of gait in a patient with delayed healing post hip fracture: a case report. Physiother Theory Pract.

[32] Kristensen MT, Bandholm T, Bencke J, Ekdahl C, Kehlet H (2009). Knee-extension strength, postural control and function are related to fracture type and thigh edema in patients with hip fracture. Clin Biomech (Bristol, Avon).

[33] Lamb SE, Morse RE, Evans JG (1995). Mobility after proximal femoral fracture: the relevance of leg extensor power, postural sway and other factors. Age Ageing.

[34] Sherrington C, Lord SR, Herbert RD (2003). A randomised trial of weight-bearing versus non-weight-bearing exercise for improving physical ability in inpatients after hip fracture. Aust J Physiother.

[35] Kronborg L, Bandholm T, Palm H, Kehlet H, Kristensen MT (2014). Feasibility of progressive strength training implemented in the acute ward after hip fracture surgery. PLoS One.

[36] Guccione AA, Fagerson TL, Anderson JJ (1996). Regaining functional independence in the acute care setting following hip fracture. Phys Ther.

[37] Kimmel LA, Liew SM, Sayer JM, Holland AE (2016). HIP4Hips (high intensity physiotherapy for hip fractures in the acute hospital setting): a randomised controlled trial. Med J Aust.

[38] Penrod JD, Boockvar KS, Litke A (2004). Physical therapy and mobility 2 and 6 months after hip fracture. J Am Geriatr Soc.

[39] Kronborg L, Bandholm T, Palm H, Kehlet H, Kristensen MT (2017). Effectiveness of acute in-hospital physiotherapy with knee-extension strength training in reducing strength deficits in patients with a hip fracture: a randomised controlled trial. PLoS One.

[40] Cheung WH, Shen WY, Dai DL, Lee KB, Zhu TY, Wong RM, Leung KS (2018). Evaluation of a multidisciplinary rehabilitation programme for elderly patients with hip fracture: a prospective cohort study. J Rehabil Med.

[41] Nordström P, Thorngren KG, Hommel A, Ziden L, Anttila S (2018). Effects of geriatric team rehabilitation after hip fracture: meta-analysis of randomized controlled trials. J Am Med Dir Assoc.

[42] Lee SY, Jung SH, Lee SU, Ha YC, Lim JY (2019). Is occupational therapy after hip fracture surgery effective in improving function?: A systematic review and meta-analysis of randomized controlled studies. Am J Phys Med Rehabil.

[43] Magaziner J, Hawkes W, Hebel JR (2000). Recovery from hip fracture in eight areas of function. J Gerontol A Biol Sci Med Sci.

[44] Magaziner J, Simonsick EM, Kashner TM, Hebel JR, Kenzora JE (1990). Predictors of functional recovery one year following hospital discharge for hip fracture: a prospective study. J Gerontol.

[45] Beaupre LA, Binder EF, Cameron ID, Jones CA, Orwig D, Sherrington C, Magaziner J (2013). Maximising functional recovery following hip fracture in frail seniors. Best Pract Res Clin Rheumatol.

[46] Lee SY, Yoon BH, Beom J, Ha YC, Lim JY (2017). Effect of lower-limb progressive resistance exercise after hip fracture surgery: a systematic review and meta-analysis of randomized controlled studies. J Am Med Dir Assoc.

[47] Latham NK, Harris BA, Bean JF (2014). Effect of a home-based exercise program on functional recovery following rehabilitation after hip fracture: a randomized clinical trial. JAMA.

[48] Kuijlaars IA, Sweerts L, Nijhuis-van der Sanden MW, van Balen R, Staal JB, van Meeteren NL, Hoogeboom TJ (2019). Effectiveness of supervised home-based exercise therapy compared to a control intervention on functions, activities, and participation in older patients after hip fracture: a systematic review and meta-analysis. Arch Phys Med Rehabil.

[49] Wu D, Zhu X, Zhang S (2018). Effect of home-based rehabilitation for hip fracture: a meta-analysis of randomized controlled trials. J Rehabil Med.

[50] González-Montalvo JI, Alarcón T, Gotor P (2016). Prevalence of sarcopenia in acute hip fracture patients and its influence on short-term clinical outcome. Geriatr Gerontol Int.

[51] Mizrahi EH, Fleissig Y, Arad M, Blumstein T, Adunsky A (2007). Admission albumin levels and functional outcome of elderly hip fracture patients: is it that important?. Aging Clin Exp Res.

[52] Press Y, Grinshpun Y, Berzak A, Friger M, Clarfield AM (2007). The effect of co-morbidity on the rehabilitation process in elderly patients after hip fracture. Arch Gerontol Geriatr.

[53] Cohn MR, Cong GT, Nwachukwu BU, Patt ML, Desai P, Zambrana L, Lane JM (2016). Factors associated with early functional outcome after hip fracture surgery. Geriatr Orthop Surg Rehabil.

[54] Mariconda M, Costa GG, Cerbasi S, Recano P, Orabona G, Gambacorta M, Misasi M (2016). Factors Predicting Mobility and the Change in Activities of Daily Living After Hip Fracture: A 1-Year Prospective Cohort Study. J Orthop Trauma.

[55] Auais MA, Morin S, Finch L (2012). A prospective 1-year study of care process and functional recovery following osteoporotic hip fractures. Osteoporosis Int.

[56] Young Y, Xiong K, Pruzek RM (2011). Longitudinal functional recovery after postacute rehabilitation in older hip fracture patients: the role of cognitive impairment and implications for long-term care. J Am Med Dir Assoc.

[57] Binder EF, Brown M, Sinacore DR, Steger-May K, Yarasheski KE, Schechtman KB (2004). Effects of extended outpatient rehabilitation after hip fracture: a randomized controlled trial. JAMA.

[58] Resnick B, Hicks G, Orwig D (201090901). Review of the impact of exercise interventions on function post hip fracture and recommendations for future interventions. Int J Disability Community Rehabilitation.

[59] Handoll HH, Sherrington C, Mak JC (2011). Interventions for improving mobility after hip fracture surgery in adults. Cochrane Database Syst Rev.

[60] Tinetti ME, Baker DI, Gottschalk M (1999). Home-based multicomponent rehabilitation program for older persons after hip fracture: a randomized trial. Arch Phys Med Rehabil.

[61] Xiao M, Wang Q, Liu T (2022). Effect of Otago exercise programme on limb function recovery in elderly patients with hip arthroplasty for femoral neck fracture. Zhong Nan Da Xue Xue Bao Yi Xue Ban.

[62] Luk JK, Chiu PK, Tam S, Chu LW (2011). Relationship between admission albumin levels and rehabilitation outcomes in older patients. Arch Gerontol Geriatr.

[63] Kristensen MT, Foss NB, Ekdahl C, Kehlet H (2010). Prefracture functional level evaluated by the New Mobility Score predicts in-hospital outcome after hip fracture surgery. Acta Orthop.

[64] Shakouri SK, Eslamian F, Azari BK, Sadeghi-Bazargani H, Sadeghpour A, Salekzamani Y (2009). Predictors of functional improvement among patients with hip fracture at a rehabilitation ward. Pak J Biol Sci.

[65] Feng L, Scherer SC, Tan BY, Chan G, Fong NP, Ng TP (2010). Comorbid cognitive impairment and depression is a significant predictor of poor outcomes in hip fracture rehabilitation. Int Psychogeriatr.

[66] Chin R, Ng B, Cheung LP (2008). Factors predicting rehabilitation outcomes of elderly patients with hip fracture. Hong Kong Med J.

[67] Mizrahi EH, Fleissig Y, Arad M, Blumstein T, Adunsky A (2008). Rehabilitation outcome of hip fracture patients: the importance of a positive albumin gain. Arch Gerontol Geriatr.

[68] Singh NA, Quine S, Clemson LM (2012). Effects of high-intensity progressive resistance training and targeted multidisciplinary treatment of frailty on mortality and nursing home admissions after hip fracture: a randomized controlled trial. J Am Med Dir Assoc.

[69] Wallengren O, Bosaeus I, Frändin K (2021). Comparison of the 2010 and 2019 diagnostic criteria for sarcopenia by the European Working Group on Sarcopenia in Older People (EWGSOP) in two cohorts of Swedish older adults. BMC Geriatr.

[70] Rosenberg IH (1997). Sarcopenia: origins and clinical relevance. J Nutr.

[71] Yoo JI, Kim JT, Park CH, Cha Y (2022). Diagnosis and management of sarcopenia after hip fracture surgery: current concept review. Hip Pelvis.

[72] Lim SK, Beom J, Lee SY, Kim BR, Chun SW, Lim JY, Shin Lee E (2020). Association between sarcopenia and fall characteristics in older adults with fragility hip fracture. Injury.

[73] Malafarina V, Malafarina C, Biain Ugarte A, Martinez JA, Abete Goñi I, Zulet MA (2019). Factors associated with sarcopenia and 7-year mortality in very old patients with hip fracture admitted to rehabilitation units: a pragmatic study. Nutrients.

[74] Landi F, Calvani R, Ortolani E (2017). The association between sarcopenia and functional outcomes among older patients with hip fracture undergoing in-hospital rehabilitation. Osteoporos Int.

[75] Avola M, Mangano GR, Testa G, Mangano S, Vescio A, Pavone V, Vecchio M (2020). Rehabilitation strategies for patients with femoral neck fractures in sarcopenia: a narrative review. J Clin Med.

[76] Oh MK, Yoo JI, Byun H, Chun SW, Lim SK, Jang YJ, Lee CH (2020). Efficacy of combined antigravity treadmill and conventional rehabilitation after hip fracture in patients with sarcopenia. J Gerontol A Biol Sci Med Sci.

[77] Seitz DP, Adunuri N, Gill SS, Gruneir A, Herrmann N, Rochon P (2011). Antidepressants for agitation and psychosis in dementia. Cochrane Database Syst Rev.

[78] Seitz DP, Gill SS, Gruneir A, Austin PC, Anderson GM, Bell CM, Rochon PA (2014). Effects of dementia on postoperative outcomes of older adults with hip fractures: a population-based study. J Am Med Dir Assoc.

[79] Rösler A, von Renteln-Kruse W, Mühlhan C, Frilling B (2012). Treatment of dementia patients with fracture of the proximal femur in a specialized geriatric care unit compared to conventional geriatric care. Z Gerontol Geriatr.

[80] Bellelli G, Guerini F, Trabucchi M (2006). Body weight-supported treadmill in the physical rehabilitation of severely demented subjects after hip fracture: a case report. J Am Geriatr Soc.

[81] Hesse S, Konrad M, Uhlenbrock D (1999). Treadmill walking with partial body weight support versus floor walking in hemiparetic subjects. Arch Phys Med Rehab.

[82] Hesse S, Werner C, Seibel H (2003). Treadmill training with partial body-weight support after total hip arthroplasty: a randomized controlled trial. Arch Phys Med Rehabil.

[83] Pohl M, Rockstroh G, Ruckriem S (2003). Immediate effects of speed-dependent treadmill training on gait parameters in early Parkinson's disease. Arch Phys Med Rehab.

[84] Baker PA, Evans OM, Lee C (1991). Treadmill gait retraining following fractured neck-of-femur. Arch Phys Med Rehabil.

[85] Resnick B, Beaupre L, McGilton KS (2016). Rehabilitation interventions for older individuals with cognitive impairment posthip fracture: a systematic review. J Am Med Dir Assoc.

[86] Smith TO, Hameed YA, Cross JL, Henderson C, Sahota O, Fox C (2015). Enhanced rehabilitation and care models for adults with dementia following hip fracture surgery. Cochrane Database Syst Rev.

